# Bariatric Surgery in the Caribbean: Is It Safe in a Low-Volume, Third World Setting?

**DOI:** 10.1155/2012/427803

**Published:** 2012-05-08

**Authors:** Dilip Dan, Yardesh Singh, Vijay Naraynsingh, Seetharaman Hariharan, Ravi Maharaj, Surujpal Teelucksingh

**Affiliations:** Department of Clinical Surgical Sciences, The University of the West Indies, St. Augustine, Trinidad and Tobago

## Abstract

Bariatric surgery is a well-recognized modality of management of obesity. In addition to obesity, it effectively controls diabetes mellitus, and hypertension. It has been recommended that bariatric surgery should be done in “designated centers” of excellence where there is a high volume of case turnover. Caribbean nations are not spared from the global spread of the obesity epidemic; however, not many patients get the benefits of bariatric surgery. This study aimed to establish that bariatric surgery could be safely and efficiently undertaken in a low-volume center outside the “designated centers” with comparable patient outcomes even in a third world setting. Though “patient numbers” generally imply better outcome, in an environment where these numbers cannot be achieved, patients should not be denied the access to surgery once good outcomes are achieved.

## 1. Introduction

Obesity has reached epidemic levels in many countries around the world [1]. The prevalence of obesity has steadily increased over the years irrespective of demographic factors such as age, sex, race, ethnicity, or educational level [2]. It is also increasing rapidly in both industrialized and developing countries [3]. Worldwide, nearly 250 million people are obese, and the WHO has estimated that in 2025, 300 million people will be obese [4]. It is a well-known fact that obesity is associated with increased morbidity and mortality.

There have been many published reports from several Caribbean nations such as Jamaica, Barbados, Trinidad & Tobago, and St. Lucia concerning the steady rise in the prevalence of obesity from primary school age through adolescence and adulthood [5–8]. A recent PAHO/WHO report suggests that more than half of the population in Trinidad & Tobago fall within the parameters of being either overweight or obese, which is indeed quite alarming [9].

The medical management of obesity has a poor track record, but bariatric surgery has demonstrated superior weight loss and dramatic improvement in comorbidities in the postoperative period.

In the developed world, bariatric surgery is usually performed at designated centers of excellence on the basis that this leads to better outcomes. However, it is debatable if bariatric surgery should be limited to such high-volume centers [10].

In addition to control of obesity, bariatric surgery is also very effective in the management of diabetes mellitus and hypertension, which commonly afflicts this population. Since the prevalence of diabetes mellitus and hypertension is also very high in the Caribbean nations, it may well be argued that bariatric surgery should be commonly available commonplace in these islands regardless of the patient turnover once results are acceptable.

With this background, this paper aims to investigate and report the safety and effectiveness of bariatric surgery in a low-volume center in a third world setting.

## 2. Methods

After necessary approval from Hospital Authorities, data regarding patients who underwent bariatric surgery in a single surgical unit (which offers the bariatric surgery service in the whole island of Trinidad & Tobago) were prospectively recorded for a period of eight years (July 2003 through June 2011) and analyzed retrospectively.

Demographic data recorded included age, gender, weight, height, and body mass index (BMI). Clinical data recorded included preoperative biochemical parameters, comorbid illnesses, drug and medication history, type of surgical procedure undertaken, surgical duration, postoperative complications, repeat procedures, hospital stay, requirement of High Dependency Unit/Intensive Care Unit admission, postoperative biochemical parameters, postoperative medication requirements, weight loss in the postoperative period, and any other serious adverse outcome including mortality if any.

All patients were seen personally by the only trained advanced laparoscopic bariatric surgeon on an individual basis. The procedure was detailed and all risks and benefits explained. The endocrinologist then coordinated the entire metabolic and cardiac evaluation with a pulmonologist and gastroenterologist used as needed. The nutritionist provided pre- and postoperative counseling to the patient and family. The services of a psychologist were used as determined by the surgeon after the initial consult. After medical clearance was obtained, the patient was again seen by the surgeon. This multidisciplinary approach was used for all patients.

Procedures were performed at the same facility (with adequate Intensive Care Unit back up facility) with a small pool of locally trained operating room staff and the same anesthetist. Patients were given heparin on induction and compression stockings placed. Prophylactic Inferior Vena Caval Filters were placed as determined to be necessary. The patients were adequately secured on the operating table and were placed supine for the bypass and in Lloyd Davis position for the sleeve and band. A surgical assistant and camera assistant were used in addition to the scrub nurse. All cases were done laparoscopically using a 6-port technique for the gastric bypass and a 5-port technique for the band and sleeve. The gastric bypass was fashioned with a 15–20 cc gastric pouch and a 120–200 cm Roux limb with a 50 cm biliopancreatic limb. An antecolic gastrojejunostomy was fashioned using a linear stapler (ETHICON) and this was tested intraoperatively with dye and air. The sleeves were done using the Echelon (ETHICON) stapler using a 38 Fr gastric tube and the band used was the Swedish band (ETHICON).

The patients were nursed on the floor (one to one nursing for 12 hours) and ambulated within 4 hours. Low molecular weight heparin was given at this time. The nurses reported directly to the surgeon who was readily available. A very low threshold for return to the operating room was practiced. A routine gastrografin study was done by the surgeon on day 1 prior to starting the liquid diet. The patient was seen in 1 week and then at 6 weeks, 3 months, 6 months, and then yearly with a metabolic screen completed at the yearly visits. Supplements used were chewable multivitamins, calcium, vitamin D, and vitamin B_12_.

## 3. Results

Two hundred and eighteen patients underwent bariatric surgery during the 8-year study period. Twenty-two patients were lost to followup. The final analysis consisted of 196 patients; 172 Roux-en-Y gastric bypass, 15 sleeve gastrectomy, and 9 gastric banding.

Age ranged from 6 to 68 years (mean 49 years). There was a female preponderance (60%) consistent with the reports of higher prevalence of obesity in this gender in the Caribbean. Preoperative body weight ranged from 79.5–234.5 kg. The BMI ranged from 32–86 kg/m^2^ (mean 49 kg/m^2^).

Comorbidities included hypertension (80%), obstructive sleep apnea (70%), diabetes mellitus (28%), significant back pain (15%), osteoarthritis (13%), polycystic ovarian syndrome (35%), and female infertility (2%).

Actual weight lost in the postoperative period ranged from 23.2 to 127.7 kg (mean 41.2 kg). Bariatric surgery is usually considered successful if more than 50% of the excess weight is lost postoperatively and maintained at that level. Of the 172 patients who underwent gastric bypass, 134 maintained an excess weight loss of greater than 50% (mean 74%) at a mean follow-up time of 3.4 years. Nineteen patients lost less than 50% of the excess weight (mean 39%). Four patients became pregnant (against advice and contraception) within 6 months after surgery and hence never lost significant weight. Fifteen patients lost up to 82% excess weight but regained weight after 1 year. This was secondary to poor patient compliance with diet, exercise, and followup.

Diabetes resolved in 52 of 61 (85%) patients with the remaining 9 patients having excellent control with decreased medications (Table 1). Most patients' diabetes resolved within the first month following surgery. Hypertension resolved in 70 of the 87 (80%) patients, and control became relatively easier in the remaining 17 (Table 1). The majority of patients had resolution of hypertension within 3 months. Sleep apnea improved in 128 of 138 (93%) patients (Table 1). All fifteen patients using continuous positive airway pressure (CPAP) machines in the preoperative period were able to discontinue its use within 1 month. Two patients with active venous ulcers had them healed in 4 months and the varicosity related edema improved after bariatric surgery. Significant improvement in the comorbidities was noted even for those patients whose weight loss was not adequate. Average length of stay for all patients ranged from 20 hours to 10 days (mean 1.9 days) with 92% patients discharged within 48 hours after surgery. Length of procedure ranged from 46 minutes to 210 minutes (mean 75 minutes).

Twelve patients underwent simultaneous cholecystectomy and 8 underwent subsequent cholecystectomy. Prophylactic ursodiol was used in 24 patients. Unexpected findings at surgery included malrotation in 2 patients and jejunal diverticulosis in 4 patients. Twelve patients had severe adhesions; these were managed prior to doing the bariatric procedure.

Blood transfusions were required in 7 patients (Table 2). Three patients had postoperative bleeding on day 1, two managed with relaparoscopy and control of the staple line with clips. The third patient was managed conservatively and settled. Four patients developed upper GI bleeding at the gastrojejunostomy site and these occurred at day 2, week 6, 7, and after 1 year. All were managed with endoscopy and cautery control.

Reoperations were performed in 7 patients (2 described above). One patient was taken back on day-2 for laparoscopy for persistent tachycardia to rule out anastomotic leak (negative). Two patients developed intestinal obstruction due to adhesions (one due to previous myomectomy and the other at the proximal Roux limb) (Table 2). Both were managed laparoscopically. Another patient developed adhesions and was managed by another surgeon with laparotomy and lysis of a single band at the jejunojejunostomy. The last patient developed a GI bleed on day 1 which caused clot obstruction at the jejunostomy and a very small leak of the remnant stomach's staple line. A laparotomy was done to correct this; the patient then developed an incisional hernia which was repaired 2 years later during the abdominoplasty.

There was no mortality in this series. Prophylactic IVC filters were used in 6 patients and 2 patients developed DVT postoperatively (one in a patient with an IVC filter at 4 months and the other at 1 month) (Table 2). Symptomatic hypoglycemia was found in 4 patients all managed with dietary changes and 1 with acarbose. Four patients had a preoperatively diagnosis of bipolar disorder and they all underwent a gastric bypass. Weight loss was excellent in 3 and average in 1 but management of the psychiatric disorder was very difficult in all with two even contemplating suicide.

All 9 patients having gastric banding lost >50% excess body weight. However, weight regain occurred in all with an average excess body weight loss of 33%. Three patients developed gastric band erosions requiring removal and conversion to sleeve gastrectomy. One patient developed an early slippage and had the band removed by another surgeon. Two of the band erosion patients developed port site infections requiring removal of the subcutaneous ports prior to band removal. All bands were placed very early in the series and this procedure has since been abandoned (Table 2).

Fifteen patients underwent sleeve gastrectomy with average followup of 8 months. Ages ranged from 6–68 years. Average weight loss is 55.4% excess weight. One patient developed a gastroparesis which required endoscopic placement of a nasojejunal tube for feeding (Table 2). This was removed after 8 days when the patient was able to swallow again. This patient had lost 27.7 kg in a 6-month period. Two patients had sleeves as the first part of a 2-stage operation (due to super morbid obesity) one of whom required to be converted to a gastric bypass.

## 4. Discussion

The major finding of the present study is that laparoscopic bariatric surgery can be performed in a low-volume center in a third world setting with low complication rates.

The American Society for Bariatric Surgery (ASBS) has proposed categorization of certain bariatric surgical practices into “Centers of Excellence” for bariatric surgery. Criteria for becoming a center of excellence include a threshold volume of bariatric surgical cases per year, operative outcomes, and the presence of a multidisciplinary commitment to management of the morbidly obese (Table 3) [11].

The relationship between volume and outcome has been established in several complex abdominal operations [12, 13]. However successful procedure outcomes can be achieved by surgeons in low volume centers [14]. The concept of centralization of surgery into specialized and superspecialized centers may not apply to less populous nations [15].

 Accreditation of Centers of Excellence in bariatric surgery requires a hospital volume of more than 125 procedures/year. Controversy exists about the perioperative safety of bariatric surgery and the relationship between volume and outcomes. There is no evidence-based rationale for this specific threshold of 125 procedures/year [16].

 There was no mortality in this series. The mortality rate from a meta-analysis of 85, 048 patients was 0.28% at 30 days after surgery and 0.35% between 30 days and 2 years [17]. The Longitudinal Assessment of Bariatric Surgery (LABS) Consortium, a 10 center prospective trial involving 4776 patients undergoing bariatric surgery, reported a 30-day postoperative mortality of 0.3% [18].

 Major complications in this series were hemorrhage, intestinal obstruction, deep vein thrombosis, band erosions, band slippage, and delayed gastric emptying (Table 2). In the multicenter LABS study of 4776 patients a major (90 day) complication rate of 4.3% was reported [18]. In our series the overall major complication rate for bariatric surgery was 16/197 (8.1%). The 90-day complication rate was 9/197 (4.6%). There was no conversion to open and the majority of operative complications were performed laparoscopically.

 Resolution of comorbidities in the present study was comparable to international data published in meta-analyses [19, 20]. Diabetes Mellitus, Hypertension and Sleep Apnea resolved in most patients in the present study (Table 1). In a recent systematic review and meta-analysis by Buchwald et al., which included 135, 246 patients, they demonstrated a 78.1% complete resolution of diabetes and diabetes was improved or resolved in 86.6% of patients [20]. In this study, diabetes mellitus resolved in 85% of patients, while the remaining 15% have excellent control on reduced medication.

Bariatric surgery decreases the prevalence of hypertension by 50% while another 25% of patients have a reduction in the number of medications or their dosage [21]. Buchwald et al. demonstrated a resolution of hypertension in 61.7% of patients [19]. In the Swedish obese subjects trial, the prevalence of hypertension decreased by 50% at 2 years (22). Therefore, a significant proportion of bariatric surgical patients show resolution of hypertension. In this study, hypertension resolved in 70/87 (80%) of patients.

Seventy percent of patients undergoing bariatric surgery has sleep apnea syndrome [22]. Bariatric surgery is effective in decreasing the severity in 100% patients with 80% of patients using CPAP able to stop treatment [23]. Sugerman et al. demonstrated in patients undergoing gastric bypass a reduction of sleep apnea syndrome from 44% to 8% between 3 to 12 months postoperatively [24]. In this study sleep apnea was improved in 95% of patients with 100% of patients weaned off CPAP machines.

 Surgery is considered successful if more than 50% of the excess weight is lost and maintained and if the comorbidities resolve. In this study excess weight loss after surgery was greater than 50%, 88.4% of gastric bypass patients, 55.4% gastric sleeve, and 33% band with followup of 3.4, 0.9, and 6.4 years, respectively. In a meta-analysis by Buchwald et al. excess weight loss after gastric bypass was 68.2% and gastric banding was 61.6% [19]. In a systematic review of sleeve gastrectomy, excess weight loss was 55.4% [25].

 Despite being a low-volume center (218 bariatric cases over 8 years) and a low-volume surgeon (27 cases per year) and not fitting the criteria for a center of excellence, we have demonstrated that bariatric surgery can be performed safely with acceptable morbidity and mortality. This is made possible by having a well-trained vigilant surgical team, thorough preoperative evaluation by a multidisciplinary team and close personalized postoperative followup by the surgeon himself for all cases.

## 5. Conclusion

Obesity is highly prevalent in the Caribbean and bariatric surgery is a safe and effective therapy for this modern epidemic. Bariatric surgery provides effective weight loss, dramatic resolution for many obesity-related diseases. This study demonstrated that bariatric surgery is safe and effective in this low-volume center in a third world setting. “Patient numbers” should not be exclusively considered as a factor to determine and/or predict safety of bariatric surgery in surgical practice. Furthermore, patients should not be deprived access to this most important treatment exclusively based on number of procedures but rather on outcome.

## Figures and Tables

**Table 1:** Resolution of comorbidities after bariatric procedures in a low-volume center.

Comorbidities	Resolution (%)
Diabetes mellitus	85.2
Hypertension	80.1
Obstructive sleep apnea	92.8

**Table 2:** Complications after Roux-en-Y gastric bypass, gastric banding, and sleeve gastrectomy.

Bariatric Proceduren (total no. of cases)	Complications	No. of patients	Percentage
Roux-en-Y gastric bypass (172)	Haemorrhage	7	4.1
	Intestinal obstruction	2	1.2
	Deep vein thrombosis	2	1.2
	Anastomotic leaks	0	0

Gastric banding (9)	Band erosion	3	33.3
	Band slippage	1	11.1
	Portinfection	2	22.2

Sleeve gastrectomy (15)	Gastroparesis	1	6.7

**Table 3:** Proposed criteria for becoming a center of excellence according to the American Society for Bariatric Surgery.

Proposed criteria for becoming a center of excellence	
Institutional commitment to in-service education program	
Perform >125 bariatric surgical cases per year	
Bariatric medical director in decision loop	
Full consultative staff and critical care services	
Full line of equipment and instruments for the care of the surgical patients	
Bariatric surgeon with 51% of practice in bariatrics	
